# Environmental Surveillance of Vector-Borne Diseases in a Non-Sewered System: A Case Study in Mozambique

**DOI:** 10.1021/acs.est.4c09860

**Published:** 2025-02-14

**Authors:** Silvia Monteiro, Filipa Nunes, Michaque Dosse, Nidia Cangi Vaz, Clemêncio Nhantumbo, Dinis Luiz Juízo, Ricardo Santos

**Affiliations:** † Laboratório de Análises, Civil Engineering Research and Innovation for Sustainability and Department of Nuclear Sciences and Engineering, Instituto Superior Técnico, Universidade de Lisboa institution, Lisboa 1049-001, Portugal; ‡ Faculdade de Engenharia, 247522 Universidade Eduardo Mondlane, Maputo 1102, Mozambique; § Centro de Biotecnologia, 54712 Universidade Eduardo Mondlane, Maputo 1102, Mozambique

**Keywords:** arboviral diseases, environmental surveillance, non-sewered settings, public health surveillance, less economically developed countries

## Abstract

Arboviral diseases pose major economic and social threats in less economically developed countries (LEDCs), where monitoring is challenging, especially in rapidly growing cities with informal settlements. In this study, we aimed to explore environmental surveillance (ES) in a non-sewered setting as a complement to syndromic surveillance in Maputo, Mozambique. Water samples were collected from nine points along the Infulene River (*n* = 66) in Maputo, Mozambique from February to September 2023. The presence of arboviruses (Dengue (DENV), Chikungunya (CHIKV), West Nile (WNV), and Usutu (USUV) virus) was determined by RT-qPCR. For the specific detection of CHIKV, two RT-qPCR assays were used: the Nsp1, targeting the non-structural protein 1 gene (*nsP1*) and the E1, targeting the E1 envelope protein gene (*E1*). DENV was detected in 82% (54/66) of the samples, with a median viral RNA load of 2.7 × 10^–2^ (2.2 × 10^5^ copies/L (cp/L)), while CHIKV was detectable in 98% (65/66) of the samples, with a median viral RNA load of 4.8 × 10^–2^ (2.2 × 10^5^ cp/L) for the *nsP1* gene and 8.0 × 10^–2^ for the *E1* gene (4.8 × 10^5^ cp/L), and USUV was detected in 6% (4/66) of the samples at a median viral RNA load of 4.1 × 10^–7^ (0 cP/L), with viral RNA load in positive samples varying between 1.8 × 10^–3^ (7.1 × 10^2^ cp/L) and 4.95 × 10^–2^ (2.1 × 10^3^ cp/L). WNV was not detected throughout the study. The prevalence and concentration varied across sampling dates. Our study demonstrated the potential of ES as a tool for assessing the circulation of arboviruses in Mozambique, where a sewered system is unavailable. Consequently, ES could be expanded from polio surveillance to include other targets in LEDCs.

## Graphical abstract

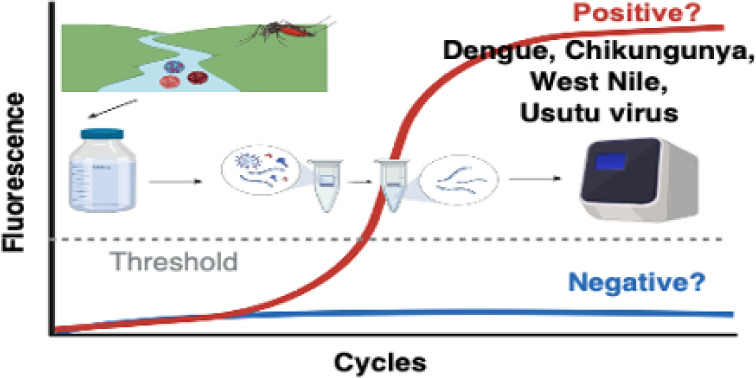

## Introduction

Arboviruses encompass a large group of viruses, including Dengue virus (DENV), Chikungunya virus (CHIKV), Zika virus, and West Nile virus (WNV), among others. These viruses and their vectors are endemic in many parts of the world and can cause serious health problems and economic losses.^1^ Due to climate change, urbanization, and globalization, the mosquitoes responsible for transmitting these viruses are now found in many previously non-endemic locations.^2^ Arbovirus circulation occurs through three different cycles: syndromic, vector, and sylvatic.

Syndromic surveillance, particularly in less economically developed countries (LEDCs), has low sensitivity and specificity, heavily relying on the reporting and severity of clinical symptoms and their overlap with other diseases in the population.^3^ For arboviruses, a significant proportion of patients are asymptomatic or experience mild, nonspecific symptoms like the flu, with estimates suggesting that over 50% of infected individuals show no symptoms.^4^ Although individual sampling and testing would theoretically be the most accurate method for tracking active transmission and disease circulation, this is impractical and economically challenging in LEDCs particularly because many people live in rural areas with limited access to urban diagnostic centers, where tests are also often unavailable.

The sylvatic and entomological cycles involve capturing animals and trapping insect reservoirs. However, using the presence of insect reservoirs as a proxy for human infections may not accurately reflect epidemiological data.^4^ These three approaches lack sensitivity and specificity, are resource-intensive, and lack spatial precision.^5^

Simple and transversal solutions are needed to complement existing surveillance programs for arboviruses. Wastewater and environmental surveillance have emerged as important tools to complement syndromic surveillance, particularly during the COVID-19 pandemic. Similar approaches have been used successfully for decades, especially in the polio eradication program, where sewage and other environmental samples play a crucial role in the surveillance, including in demonstrating the efficacy of vaccination programs.^6^

It is important to determine whether ES could be helpful in identifying infection trends in populations without centralized sewer systems, complementing clinical and vector surveillance.^7^ On one hand, arboviral RNA has been shown to be excreted in the urine of infected patients.^8^ On the other hand, research indicates that DENV RNA can persist for several days at temperatures up to 37 °C, suggesting that monitoring viral RNA in environmental waters could complement clinical surveillance for arboviral diseases.^9^

In this study, we aimed to test the use of ES in a non-sewered system for a range of arboviral diseases in Maputo, Mozambique.

## Materials and Methods

### Study Design

This surveillance study was conducted using samples collected from the Infulene River in Maputo, Mozambique. We validated a previously published hydrolysis probe-based RT-qPCR assay designed to detect WNV and Usutu virus (USUV), while assays for detecting DENV and CHIKV had been tested previously.^10^ These assays were employed to determine the presence of different arboviruses in water samples from the Infulene River basin over an eight-month period.

We measured the levels of these arboviruses in environmental waters because clinical surveillance is sparse in sub-Saharan Africa, and febrile patients are often presumed to have malaria and are treated accordingly. However, many of these patients test negative for malaria, as shown by the only study reporting the presence of DENV, CHIKV, and WNV in febrile patients in Mozambique thought to have malaria.^11^ The Infulene River spans a total area of 147 km², with a main stream approximately 20 km long. The Infulene River is situated in the Infulene Valley, on the periphery of the city of Maputo, Mozambique. It is an area of great social, economic, and environmental importance. The community around the Infulene River is a community of small-scale farmers engaged in vegetable production, family owned and labor dependent farmland that produces food for local markets and for families’ own consumption.^12^ The community suffers from a number of socioeconomic problems, such as the discharge of untreated wastewater into the river which contaminates water sources for irrigation while exposing farmers and consumers to health hazards.^13^ The rapid urbanization and industrialization in the region put agricultural water supplies under pressure, uprooting farmers, further worsening their socioeconomic problems.^14^ The Infulene River Basin experiences distinct rainy (October–March) and dry (April–September) seasons, with mean annual rainfall ranging from 400 to 1000 mm. The mean annual temperature varies from 22 °C to 29 °C, and relative air humidity ranges between 67.3% and 80.5%.

In the Municipality of Maputo, the sewer network consists of two distinct systems: system 1, built in the 1940s, operates as a combined sewer, discharging directly into the bay, and system 2, constructed in the 1980s, that includes sewer lines, a wastewater treatment plant (WWTP), and two pumping stations. However, the pumping stations in system 2 are not currently operational.^15^ High population density in the informal settlements with very poor sanitation systems, coupled with a lack of resources for constructing improved latrines and a shallow water table, leads to the direct discharge of domestic wastewater into drainage ditches. This causes microbiological contamination, attracts insects, and results in unpleasant odors.^16^

The Infulene River Basin is a good example of a river that is extremely vulnerable to contamination not only by wastewater coming from two wastewater treatment plants and contaminated drainage water coming from informal settlements in Maputo City, but also from contaminants leaching from agriculture field where chicken manure is used as fertilizers. Additionally, understanding the level of contamination of the water of the river is important because it is used for urban agriculture that is developed along the river basin wetlands which is the main source of vegetables to Maputo and Matola Cities.

### Sample Collection

Water samples (*n* = 66) were collected once a month from five points along the Infulene River basin and four open drain points (Figures 1 and S1 and Table S1) between February 17 and September 6, 2023. The sampling points had been chosen previously, considering the potential entrance of contamination in the Infulene River, beingselected based on their representativeness and accessibility.^17^ Point 9 marks the start of the Infulene River. Point 8, an open drain, was chosen because it receives input from the Zimpeto WWTP, which was and still currently is not operational. Point 7, located along the Infulene River, receives water from the surrounding neighborhoods and also receives input from the open drain that feeds into Point 8. Point 6 is the last sampling point in the Infulene River before the flow from the open drain along Joaquim Chissano Avenue enters the river. Point 5 was taken from the open drain running along Joaquim Chissano Avenue, which is influenced by nearby neighborhoods. In contrast, point 4 is a highly contaminated open drain of unknown origin, which merges with the Joaquim Chissano Avenue drain downstream of point 5 at point 3, an open drain, which then joins the Infulene River. Finally, points 2 and 1 are located in the Infulene River just before and after the discharge from the Infulene WWTP, respectively, which was operating on a very low removal efficiency. During the sampling period, the Infulene WWTP was clogged, and due to a blockage of sludge—primarily caused by inert material—the removal efficiencies were significantly lower than under normal operational conditions with fully functional ponds. The samples were taken in duplicate at a depth of 10–15 cm and stored in 1-liter sterile plastic containers. They were then refrigerated and transported to the Environmental Engineering Laboratory at the Chemical Engineering Department of Eduardo Mondlane University, where they were frozen at −30 °C until shipment to Lisbon, Portugal. During transport, the samples remained frozen and were stored at −80 °C upon arrival in Lisbon until further processing. This study did not require ethical oversight as there was no direct human involvement and the results were anonymized.

**Figure 1:**
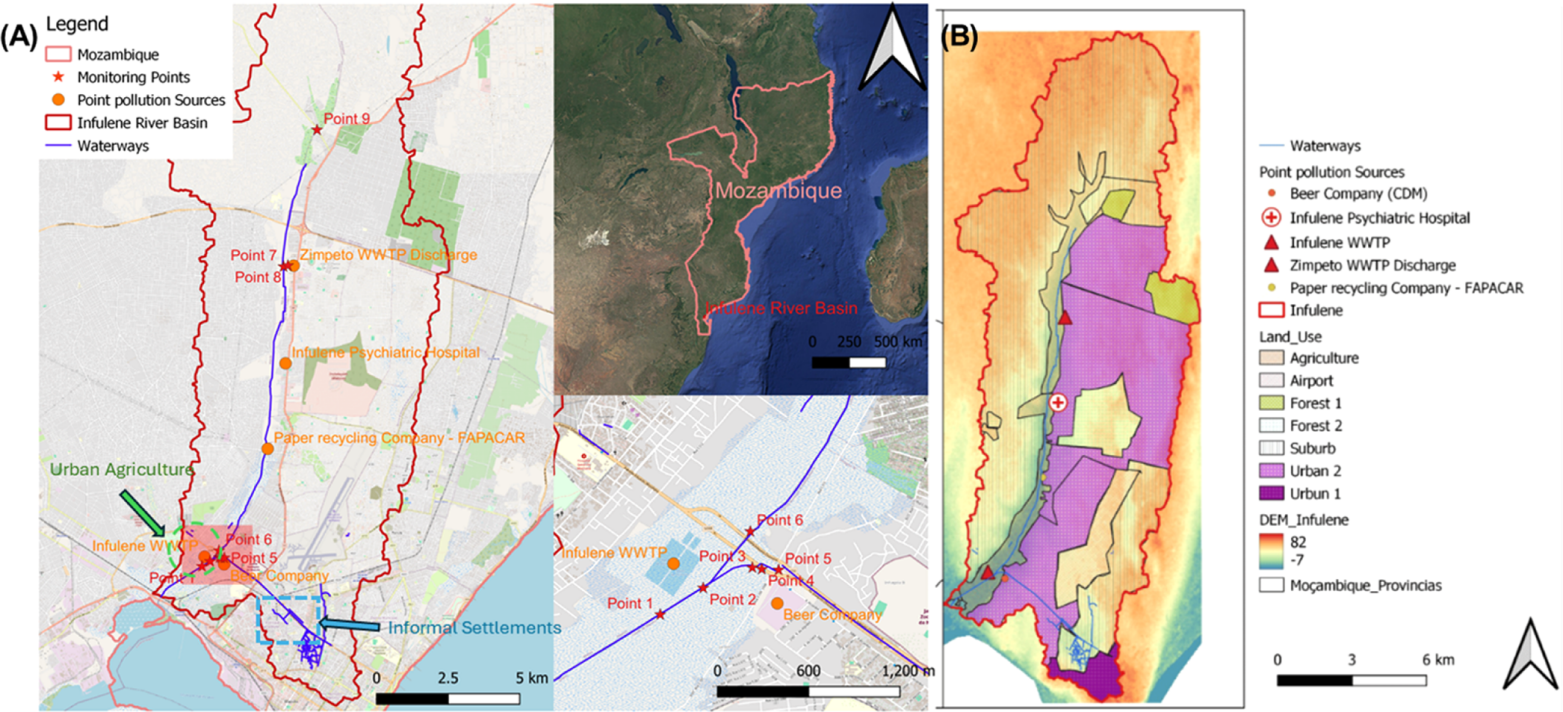
Study area and sampling points (A). Location of point and non-point sources of contamination.^17^ WWTP—Wastewater treatment plant.

### Processing of River Water

To concentrate the samples, direct precipitation with polyethylene glycol 8000 (PEG 8000) was performed following a modified version of Monteiro et al. (see Supporting Information).^18^ In brief, a viral stock of porcine epidemic diarrhea virus (PEDV) was added, to each sample, as a process control to a final concentration of 1.21 × 10^4^ copies/L (quantified by RT-dPCR as described in Supporting Information). PEG 8000 (Sigma-Aldrich, Steinheim, Germany) was added to a final concentration of 20% (w/v) to 300 mL of river water, and the samples were precipitated overnight at 4 °C. The samples were then centrifuged at 10,000 × *g* for 30 min, followed by resuspension in 5 mL of 1× PBS at pH 7.4. The samples were stored at −80 °C ± 10 until further processing.

### Viral RNA Extraction, Detection, and Quantification

Nucleic acid extraction was performed on 220 μL of concentrated samples using the QIAamp FAST DNA Stool Mini kit (QIAGEN, Hilden, Germany), with a final elution volume of 100 μL, following the manufacturer’s instructions (Supporting Information). The RNA extraction recovery efficiency was assessed using murine norovirus (MNV).

The primers and probes used in this study are listed in Table S2. One-step RT-qPCR assays (Luna Universal Probe One-Step RT-qPCR, New England Biolabs, Massachusetts, USA) were employed to detect DENV, CHIKV, WNV, USUV and Pepper Mild Mottle Virus (PMMoV). PMMoV is found consistently worldwide and has been previously utilized to normalize wastewater and environmental surveillance data, accounting for variations in flow.^19,20^ The detection of CHIKV was conducted using two target genes: the non-structural protein 1 (*nsP1*) and the E1 envelope protein (*E1*). To detect viral nucleic acids, 4-fold dilutions of each viral extract were assayed alongside crude extracts to mitigate potential amplification inhibition due to the complex nature of the samples. Details of the RT-qPCR protocols are provided in Supporting Information.

All amplification reactions were conducted using a QuantStudio 5 Real-Time PCR System (ThermoScientific, Massachusetts, USA). Quantification of DENV and CHIKV was achieved using calibration curves with 5- and 10-fold dilutions of the TaqMan Arbovirus Triplex Control Kit (ZIKV/DENV/CHIKV) (ThermoScientific, Massachusetts, USA), ranging from 6.0 to 6.0 × 10³ and 1.0 to 1.0 × 10^4^ copies per reaction, respectively. Standard curves for WNV and USUV were constructed using gBlocks® Gene Fragments (Integrated DNA Technologies, IA, USA) as detailed in Supporting Information. Performance details of the standard curves are provided in Supporting Information. Negative controls, including extraction and RT-qPCR assays, were conducted using DNase/RNase-free water (Table S3 and Figure S2).

### Validation of Arboviral Detection in River Water

The validation of the results and the selection of the WNV and USUV assays were conducted in multiple stages: (i) in silico, by assessing the assays’ performance against the GenBank database using the Basic Local Alignment Tool (BLAST; available from URL: http://www.ncbi.nlm.nih.gov/BLAST/); (ii) in situ, using q­(RT)­PCR against microorganisms commonly found in river water (Table S4); (iii) through amplicon sequencing.

For the amplicon sequencing, samples that tested positive after RT-qPCR were run on a 4.0% agarose gel, and the results were visualized after electrophoresis and ethidium bromide staining. Bands of the correct size (Table S2) were excised from the gel and extracted using the EXTRACTME DNA clean-up gel-out kit (Blirt, Gdańsk, Poland), following the manufacturer’s instructions. The DNA was recovered in a final volume of 50 μL. Positive controls for both viruses were also sequenced in parallel with the samples. Sequencing was conducted by an external laboratory (StabVida, Lisbon, Portugal). The magnetic beads-purified DNA amplicons were sequenced using the ABI 3730xl (ThermoScientific, Massachusetts, USA).

Upon receiving the sequences, the chromatograms were visualized using Chromas (version 2.6.6) to inspect the quality of the sequences. Sequences were trimmed, and those with reliable sequence data, with less than 5% of bases identified as ambiguous, were used for further evaluation. After quality control, the reads were aligned with the GenBank database using BLAST, and the NCBI Multiple Sequence Alignment Viewer was used to obtain a graphical display for nucleotide sequence alignments.

### Statistical Analysis

Data analysis was performed using SPSS version 26 (IBM Corporation, New York, USA) and R (version 4.2.2). Figures were generated with RStudio, utilizing the ggplot2 package (version 3.4.4).

All RT-qPCR data were converted into a logarithmic format by adding 1 to each value, following normalization with PMMoV and subsequent log10 transformation. The normality of the data was assessed using the Shapiro-Wilk test and the Q-Q plot. The Kruskal-Wallis test was employed to evaluate variations in viral RNA loads across different sampling points and campaigns. The Spearman rank correlation level and respective 95% confidence intervals (95% CI) were calculated to determine potential associations between viral RNA load of the most commonly detected viral targets (DENV, CHIKV *nsP1* gene, and CHIKV *E1* gene). Data compatibility was determined using the S-value, also known as the Shannon information or surprisal, with p-values provided for context. p-values below 0.05 were considered statistically significant. The S-value was calculated according to eq 1:^21^

1 $$s=-log_{2}\left(p\right)$$

## Results and Discussion

The control experiments yielded the anticipated results, including both the negative and positive extraction and amplification controls. PEDV recovery efficiencies ranged from 36% to 72%, while extraction efficiency using MNV as a proxy was 68% (±10%). All samples showed positive results for both the process and extraction controls. However, in the eventuality that any of the samples yielded unsatisfactory control results, the following actions would be taken: (i) if PEDV was not detected in the samples, re-extraction would occur only if the extraction control indicated low levels or was absent; (ii) if the extraction control yielded satisfactory results, the sample would be excluded from the dataset; (iii) if the extraction control was absent, the samples would be re-extracted, and if the issue persisted, they would also be excluded from the dataset.

In silico analysis showed no cross-reactivity of the selected assays with sequences in the NCBI database. The assays were tested in vitro against non-target gDNA and gRNA (Table S4), and no cross-reactivity was observed (Table S5).

DENV and CHIKV RNA was consistently detected throughout the study in the Infulene river basin (Figure 2). In contrast, USUV was detected only during the first and third sampling campaigns, and WNV was not detected at all. Specifically, DENV was found in 54 out of 66 samples (82%, see Supporting Information on how to calculate the percentage of positive samples). CHIKV was detected in 65 out of 66 samples (98%), while USUV was detected in 4 out of 66 samples (6%).

**Figure 2:**
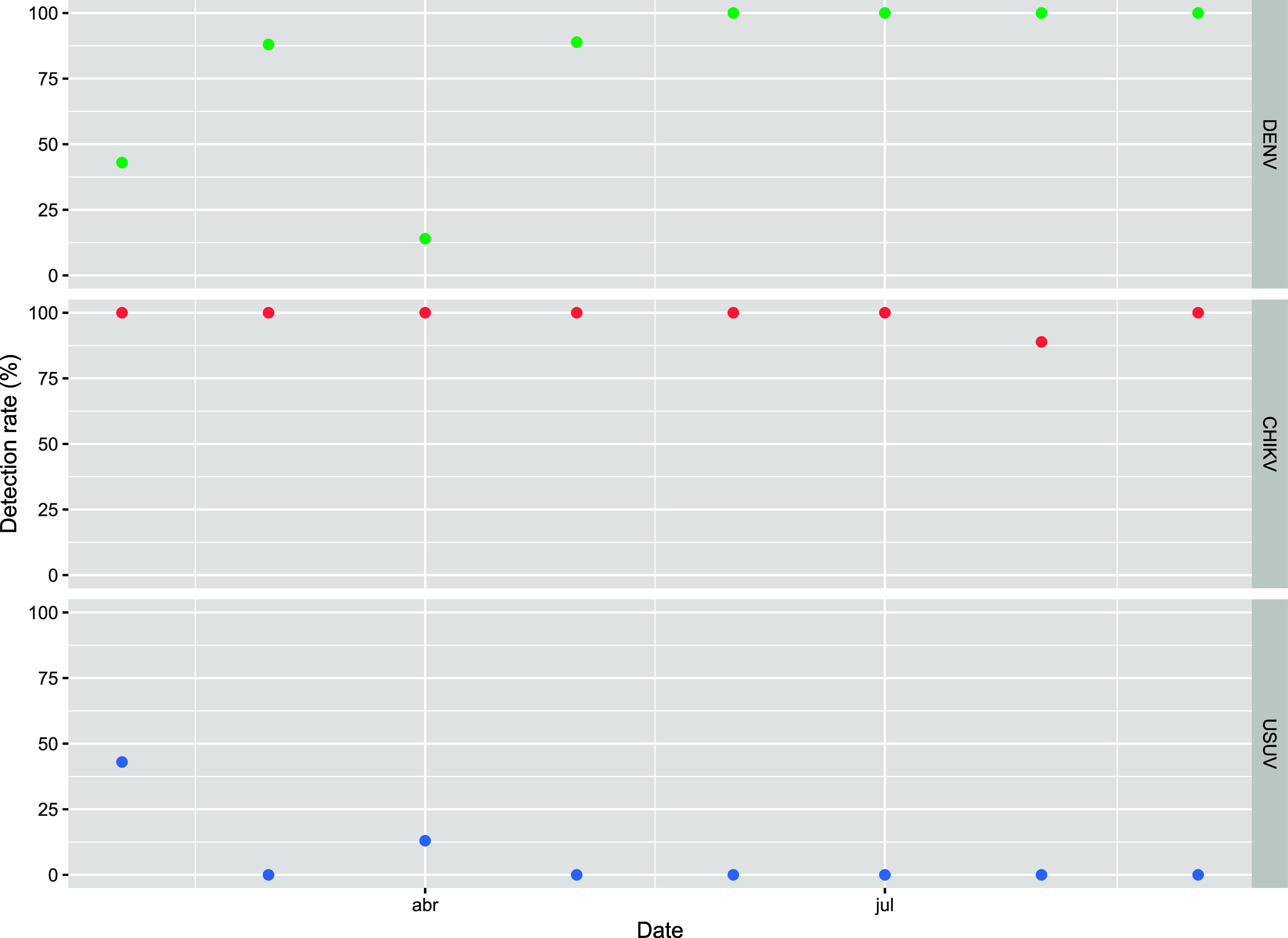
Detection rate of DENV, CHIKV, and USUV in the Infulene river basin in Maputo, Mozambique. For the detection rate of CHIKV, the samples where both genes were detected were counted as a single sample. Measurements were conducted between February 17, 2023 and September 6, 2023. Detection rate (%) = # positive samples × 100/total # of samples. DENV = Dengue virus. CHIKV = Chikungunya virus. USUV = Usutu virus.

To conduct ES, it is essential to identify the most suitable sampling locations (Figure 3). In our study, sampling points 1 and 5 generally showed the highest detection rates for DENV and CHIKV (*nsP1* and *E1* genes), with USUV detected in 1 out of 7 samples (14%) at point 1 and 1 out of 8 samples (13%) at point 5. Point 9 also displayed high detection rates for DENV (8 out of 9 samples (88%)) and CHIKV (*nsP1* gene: 9 out 9 samples (100%); *E1* gene: 8 out 9 samples (88%)); however, we were unable to retrieve USUV in this point. In contrast, points 7 and 8 exhibited lower detection frequencies for DENV (point 7:7 out of 8 samples (88%); point 8:5 out of 8 samples (63%)) and CHIKV (*nsP1* gene point 7:6 out of 8 samples (75%), point 8:7 out of 8 samples (88%); *E1* gene point 7:5 out of 8 samples (63%), point 8:6 out of 8 samples (75%)), although USUV was detected in 1 out of 8 samples (13%) at point 8. In fact, when combining the samples positive for both CHIKV genes, CHIKV was detectable in all locations in all sampling campaigns, with the exception of point 7, where we were unable to detect both CHIKV genes in the August 2023 campaign.

**Figure 3:**
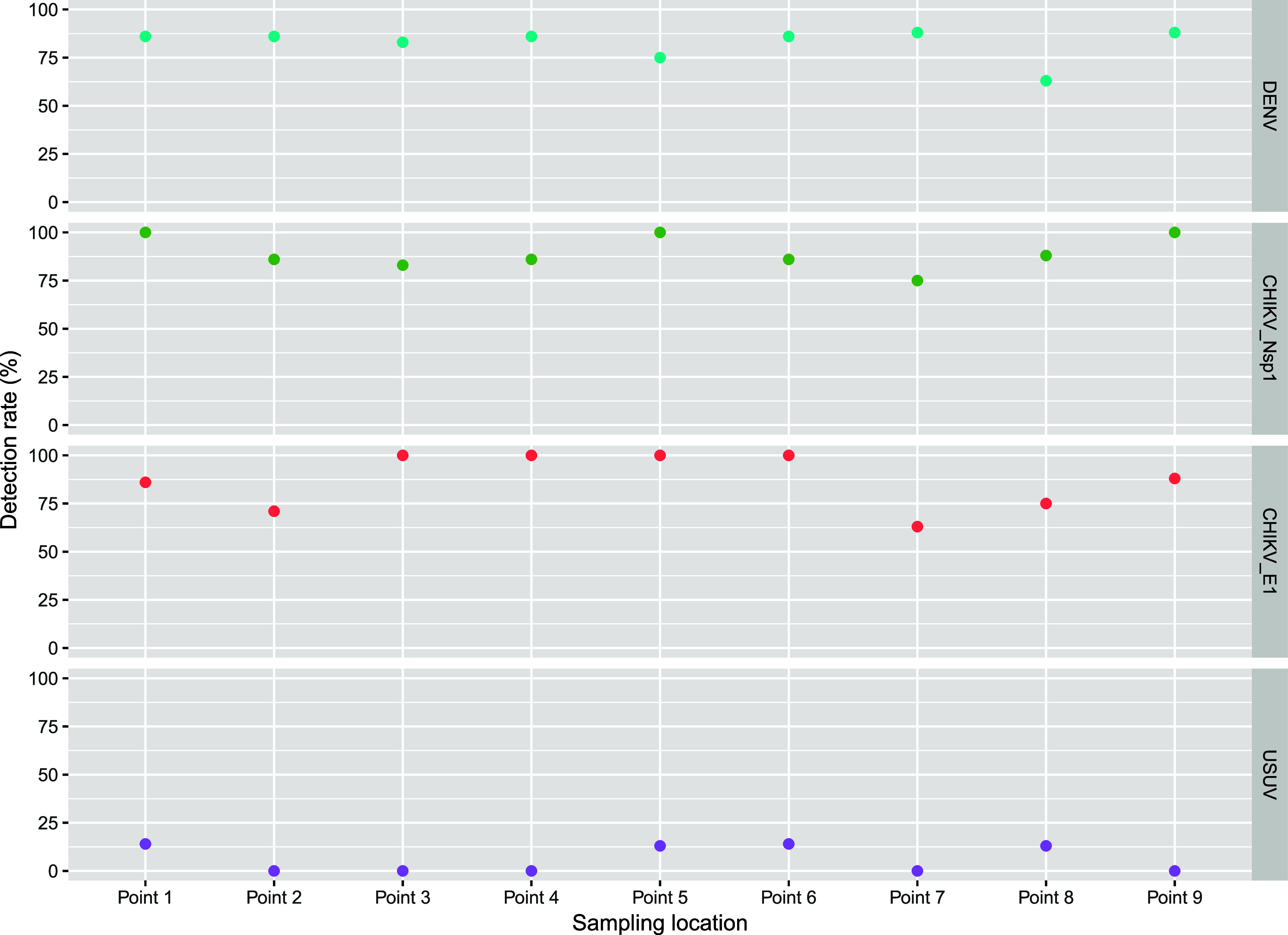
Detection rate of DENV, CHIKV, and USUV at each sampling point of the Infulene river basin in Maputo, Mozambique. For CHIKV, the detection rate for each gene (*Nsp1* and *E1*) are shown. Measurements were conducted between February 17, 2023 and September 6, 2023. Detection rate (%) = # positive samples × 100/total # of samples. DENV = Dengue virus. CHIKV = Chikungunya virus. USUV = Usutu virus.

All samples tested positive for at least one of the targeted arboviral RNAs. The majority of samples (41 out of 66 (62%)) were positive for DENV and both genes targeting CHIKV, particularly during the last three sampling campaigns. The first three campaigns exhibited a broader variety of arboviral distribution. USUV was detected only in conjunction with CHIKV (four out of 66 samples (6%). Only three samples (5%) had a single arboviral RNA gene detected. We observed a high association between the RNA viral loads of the *Nsp1* gene and the *E1* gene used for the quantification of CHIKV (*r* = 0.60 [95% CI 0.41–0.74], *p* < 0.001), a low association between DENV and the CHIKV *E1* gene (*r* = 0.31 [95% CI 0.07–0.52], *p* = 0.01) whereas no association was found between DENV and the CHIKV *Nsp1* gene (*r* = 0.19 [95% CI −0.06–0.42], *p* = 0.12).

The median viral RNA load ranged from 2.7 × 10^–2^ (interquartile range (IQR) 3.6 × 10^–3^–7.3 × 10^–1^) for DENV to 8.0 × 10^–2^ (IQR 1.0 × 10^–1^–1.7) for the CHIKV *E1* gene (see Figure 4and Table 1). The viral RNA load for DENV showed greater variability during the first three sampling campaigns, with the highest viral RNA load for the entire study recorded in March 2023. After that, the DENV viral RNA load remained relatively consistent for the remainder of the study. For CHIKV, the highest viral RNA loads were observed in March (*E1* gene) and April (*nsP1* gene), followed by a decline in May and June 2023. The CHIKV viral RNA load remained fairly stable for the rest of the study period.

**Figure 4:**
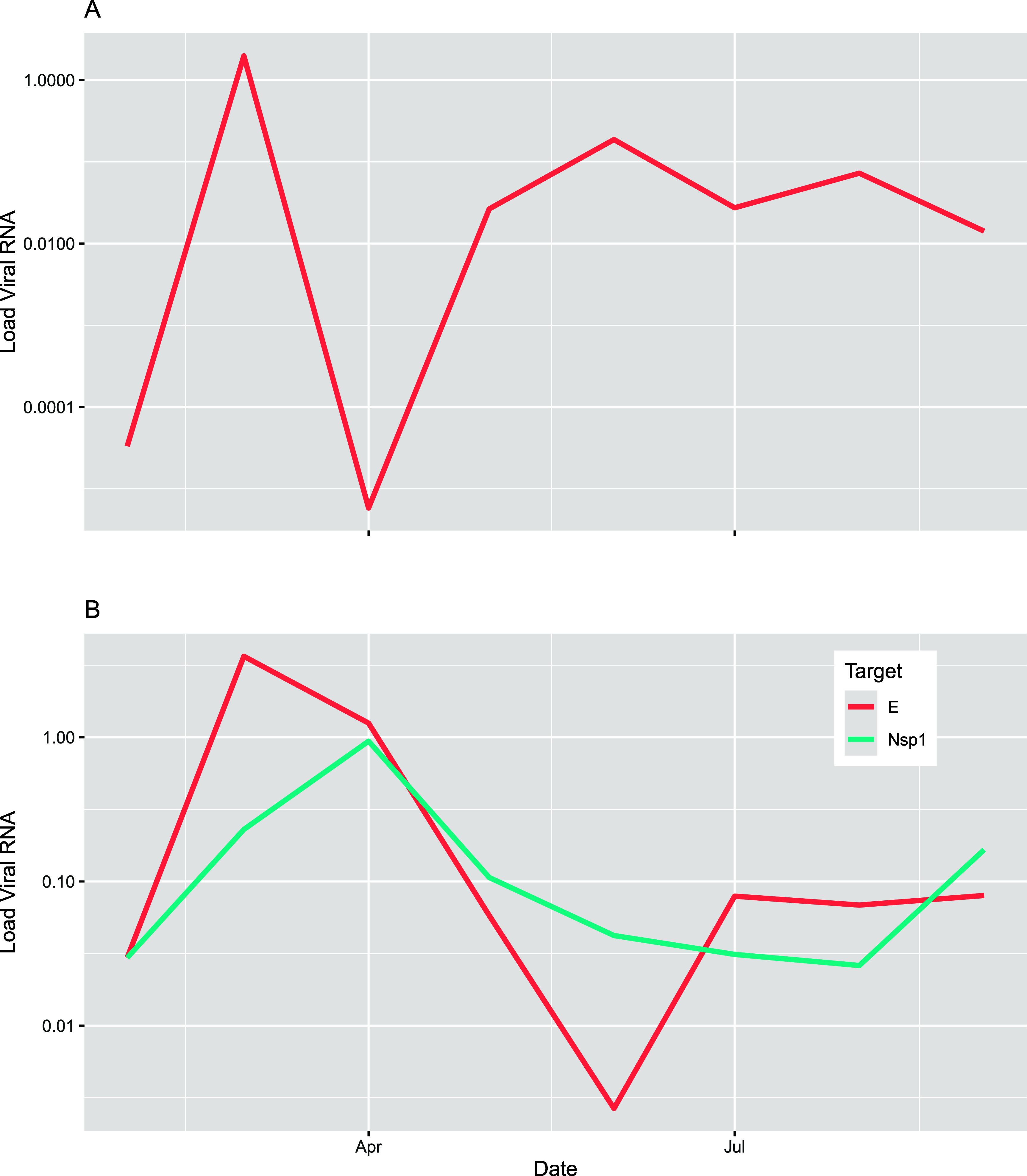
Aggregated median Load Viral RNA of DENV and CHIKV in the Infulene River Basin. Report of monthly measurements. (A) Load Viral RNA of DENV. (B) Load Viral RNA of CHIKV (expressed by the nsP1 and E1 genes). Data aggregate the concentrations measured at each sampling location for each sampling collection date. Measurements were conducted between February 17, 2023 and September 6, 2023. DENV = Dengue virus. CHIKV = Chikungunya virus.

**Table 1:** Summary Statistics of the Detection of DENV, CHIKV, and USUV in Water Samples from the Infulene River in Maputo, Mozambique^a^ ^b^ ^c^ ^d^ ^e^

	Positive samples (%)	Median load viral RNA	IQR load viral RNA
Infulene River Basin			
DENV	54 (82%)	2.7 × 10^–2^	3.6 × 10^–3^–7.3 × 10^–1^
CHIKV – Nsp1	58 (88%)	4.8 × 10^–2^	9.1 × 10^–3^–9.1 × 10^–1^
CHIKV – E	57 (86%)	8.0 × 10^–2^	1.0 × 10^–1^–1.7
USUV	4 (6%)	4.1 × 10^–7^	8.6 × 10^–8^–5.4 × 10^–6^ – 0
Sampling Point 1			
DENV	6 (86%)	2.6 × 10^–2^	5.9 × 10^–3^– 1.0 × 10^–1^
CHIKV – Nsp1	7 (100%)	1.1 × 10^–1^	7.7 × 10^–2^–5.0 × 10^–1^
CHIKV – E	6 (86%)	4.7 × 10^–1^	7.6 × 10^–1^–1.0
USUV	1 (14%)	2.2 × 10^–7^	1.6 × 10^–7^–2.2 × 10^–6^
Sampling Point 2			
DENV	6 (86%)	1.0 × 10^–1^	1.1 × 10^–2^–1.8 × 10^–1^
CHIKV – Nsp1	6 (86%)	4.5 × 10^–1^	1.6 × 10^–2^–2.6 × 10^–1^
CHIKV – E	5 (71%)	5.8 × 10^–2^	1.7 × 10^–2^–3.7
USUV	0 (0%)	2.0 × 10^–7^	1.4 × 10^–7^–2.9 × 10^–6^
Sampling Point 3			
DENV	5 (83%)	6.1 × 10^–3^	1.7 × 10^–3^–8.8 × 10^–3^
CHIKV – Nsp1	4 (67%)	2.1 × 10^–1^	2.4 × 10^–3^–1.6 × 10^–1^
CHIKV – E	6 (100%)	2.7 × 10^–2^	4.3 × 10^–3^–8.9 × 10^–2^
USUV	0 (0%)	5.6 × 10^–8^	3.9 × 10^–8^–7.5 × 10^–8^
Sampling Point 4			
DENV	6 (86%)	9.4 × 10^–2^	2.7 × 10^–2^–9.1 × 10^–1^
CHIKV – Nsp1	6 (86%)	9.7 × 10^–2^	2.0 × 10^–2^–7.7 × 10^–1^
CHIKV – E	7 (100%)	1.2 × 10^–1^	5.1 × 10^–2^–1.2
USUV	0 (0%)	9.6 × 10^–7^	5.0 × 10^–7^–1.8 × 10^–6^
Sampling Point 5			
DENV	6 (75%)	1.4 × 10^–2^	1.9 × 10^–3^–1.2 × 10^–1^
CHIKV – Nsp1	8 (100%)	6.9 × 10^–2^	2.8 × 10^–2^–2.7
CHIKV – E	8 (100%)	2.4 × 10^–1^	5.8 × 10^–2^–4.7
USUV	1 (13%)	3.0 × 10^–7^	1.0 × 10^–7^–1.4 × 10^–5^
Sampling Point 6			
DENV	6 (86%)	1.3 × 10^–1^	4.3 × 10^–2^–5.4 × 10^–1^
CHIKV – Nsp1	6 (86%)	2.4 × 10^–2^	4.1 × 10^–3^–6.9 × 10^–1^
CHIKV – E	7 (100%)	4.7 × 10^–1^	5.6 × 10^–2^–9.0
USUV	1 (14%)	8.2 × 10^–7^	1.7 × 10^–7^–1.5 × 10^–5^
Sampling Point 7			
DENV	7 (88%)	2.9 × 10^–1^	2.1 × 10^–2^–2.0
CHIKV – Nsp1	6 (75%)	2.9 × 10^–1^	7.0 × 10^–3^–1.8
CHIKV – E	5 (63%)	4.9 × 10^–1^	6.9 × 10^–3^–2.6
USUV	0 (0%)	1.8 × 10^–6^	6.9 × 10^–7^–8.1 × 10^–6^
Sampling Point 8			
DENV	5 (68%)	2.4 × 10^–3^	2.1 × 10^–7^–1.1 × 10^–2^
CHIKV – Nsp1	7 (88%)	1.5 × 10^–2^	1.3 × 10^–3^–2.8 × 10^–2^
CHIKV – E	6 (75%)	7.6 × 10^–3^	1.4 × 10^–3^–1.6 × 10^–2^
USUV	1 (13%)	7.4 × 10^–8^	1.5 × 10^–8^–2.1 × 10^–7^
Sampling Point 9			
DENV	7 (88%)	1.9	1.2–3.4
CHIKV – Nsp1	8 (100%)	2.6	6.2 × 10^–1^–5.0
CHIKV – E	7 (88%)	3.9	3.2 × 10^–1^–6.6
USUV	0 (0%)	5.8 × 10^–6^	3.8 × 10^–6^–1.8 × 10^–5^

a Prevalence, viral RNA load, and IQR of DENV, CHIKV, and USUV in the Infulene River Basin.

b DENV = Dengue virus.

c CHIKV – Nsp1 = Chikungunya–nonstructural viral protein 1.

d CHIKV – E1 = Chikungunya–envelope protein 1.

e USUV = Usutu virus.

The viral RNA load of DENV was influenced by the sampling campaign (s = 7.4 (*p* = 0.006)). Conversely, the CHIKV *nsP1* and *E1* gene, and USUV were not influenced by the sampling campaign (CHIKV *Nsp1*: s = 0.18 (*p* = 0.88); CHIKV *E1*: s = 3.3 (*p* = 0.10); USUV: s = 1.2 (*p* = 0.06)). The seasons (dry and rainy) did not influence the viral RNA load of DENV and CHIKV (DENV: s = 0.60 (*p* = 0.66); CHIKV *nsP1*: s = 0.81 (*p* = 0.57); CHIKV *E1*: s = 2.8 (*p* = 0.14)), but had influence on the viral RNA load of USUV (s = 1.7 (*p* = 0.02)).

The median viral RNA load was systematically higher for DENV and CHIKV in sampling point 9 (Figure 5and Table 1). On the other hand, the viral RNA load was lower in sampling point 8, followed by point 3, which also goes in agreement with the detection rates found for each site. The sampling location along the Infulene River basin significantly impacted the results for DENV (s = 5.06 (*p* = 0.03)), but not CHIKV, and USUV (CHIKV – *nsP1*: s = 4.06 (*p* = 0.06); CHIKV – *E1*: s = 3.64 (*p* = 0.08); USUV: s = 0.74 (*p* = 0.42)).

**Figure 5:**
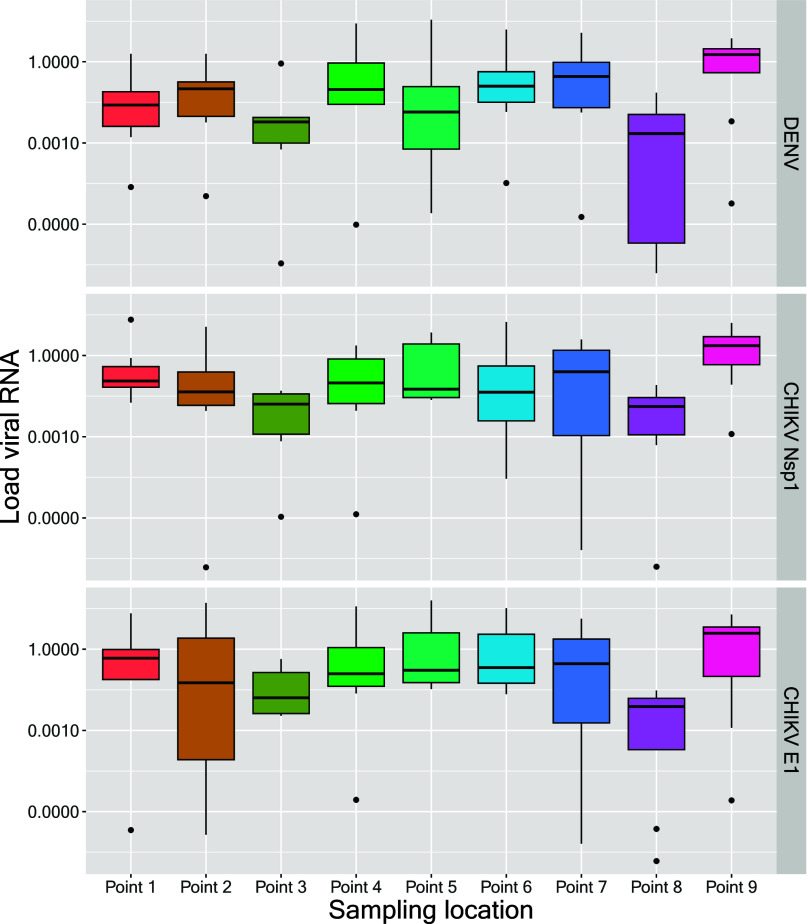
Concentration of DENV and CHIKV (nsP1 and E1 genes) for each sampling location. Boxes, 25^th^ and 75^th^ percentile; lines within the boxes, media; whiskers. 10^th^ and 90^th^ percentile, respectively; *n* = 66; Measurements were conducted between February 17, 2023 and September 6, 2023. DENV = Dengue virus. CHIKV = Chikungunya virus.

The sequencing data, despite the small fragment size preventing lineage discrimination and the creation of a phylogenetic tree, confirmed the presence of USUV in the positive samples (Figure S4). The sequence homology for USUV ranged from 97% to 100%, with trimmed sequences varying in size between 66 and 95 bp.

To our knowledge, this is the first study to detect DENV, CHIKV, and USUV in river water samples, and the first to report USUV circulation in Mozambique, as it has not been tested clinically. Throughout the study, DENV and CHIKV were consistently found in water samples from the Infulene river basin in Maputo, Mozambique, while USUV was detected less frequently; WNV remained undetected. Co-occurrence of arboviruses was observed, particularly DENV and CHIKV, as well as CHIKV and USUV. Despite a pilot study in Nepal—an endemic region for DENV—failing to detect these viruses in river water, our findings indicate that they co-circulate in Maputo, Mozambique, as described in other parts of the world.^22,23^ This co-circulation, combined with the presence of the vectors, promotes simultaneous infections in human populations. A serological study of febrile patients in Maputo revealed multiple cases of concurrent arboviral infections, and some patients were also found to have simultaneous infections with *Plasmodium* spp.^11^ In our study, CHIKV was found to be more prevalent than the other arboviruses tested, with DENV following in prevalence. This finding is consistent with Gudo et al. (2016), which reported a higher prevalence of CHIKV infections compared to DENV.^11^ The vectors for these arboviruses are present in Mozambique, suggesting a potential risk for infection despite limited knowledge on the viruses’ circulation in vectors, the sylvatic cycle, and humans due to inadequate surveillance.^24,25^ The lack of clinical surveillance data in Mozambique prevents us from linking viral RNA in environmental waters with clinical findings. Once such data become available, it will be essential to determine whether environmental trends align with clinical surveillance results. Moreover, the broad RT-qPCR assay for the different arbovirus, which does not differentiate between serotypes and lineages, adds complexity to comparing with clinical data. Another limitation from this study, due to budgetary constraints, was the sampling period, which only encompassed eight months. A shorter sampling period may have limited outbreak and long-term trends overview, does not allow to see seasonal variations, and may not allow for the detection at low-level transmission, which can translate in data interpretation challenges. However, due to the lack of clinical or entomological surveillance in Mozambique, the study underscores the importance of environmental surveillance as a valuable complement to traditional vector and clinical data, especially in the absence of effective clinical surveillance. Environmental monitoring can provide critical insights into arboviral infection trends, potentially guiding targeted clinical surveillance and other necessary measures. Comparatively, research in Lisbon, Portugal, and Miami-Dade County, US, which also examined DENV, reported lower prevalence levels than those observed in our study.^10,26^ Similarly, Portugal showed lower prevalence of CHIKV.

An important factor when performing environmental surveillance is the choice of sampling locations. Therefore, considering the number of sampling points across the Infulene river basin and the specific characteristics of each location, this study highlights the importance of selecting appropriate sampling sites. The locations were chosen based on a previous study that considered both the representativeness of the basin and accessibility.^17^ Our study demonstrates that sampling points 1, 5, and 9 are sufficient for studying arbovirus circulation in the Infulene river basin. Point 9, located near the river’s source, represents the population from that area. Point 5, situated along the open drain on Joaquim Chissano Avenue, receives inputs from a large surrounding area, while point 1, located downstream from the Infulene WWTP discharge, captures inputs from the upper part of the river and surrounding open drains. Our study highlights the critical role of ES in areas with open drains and/or lacking well-functioning WWTP. While clinical data on arbovirus infections was unavailable in Maputo, our findings could be extended to other LEDCs, where clinical surveillance may be insufficient or ineffective due to the high prevalence of asymptomatic cases, provided the catchment areas are properly studied. The results of our study aligned with the expected patterns of infection seasonality in Mozambique, demonstrating the effectiveness of ES for monitoring arboviral diseases. During our research, we identified optimal sampling locations, particularly those where multiple parts of the city converge, such as the open drainage system, to obtain a more representative snapshot of the entire population. The selection of sampling locations in open drains and river water should adhere to several key principles. These include prioritizing areas from densely populated neighborhoods, urban zones, and informal settlements with inadequate sanitation infrastructure. Sampling sites should also be located at the confluence of multiple drains or waterways, preferably downstream, to capture a wider range of contaminants. Additionally, the sites must be easily accessible and safe for fieldworkers. It’s also crucial to select locations across different geographic areas to account for the diversity within the catchment. Special attention should be given to sites near stagnant water, potential mosquito breeding grounds, or areas with high vector activity, as these are particularly relevant for monitoring arboviral diseases. Nonetheless, the choice of sampling locations, such as focusing on open drains and river water rather than fecal sludge, comes with both advantages and disadvantages. The main advantages include accessibility as these locations are widely accessible in urban and peri-urban areas, the coverage of large areas, representing a large section of the population, including areas without household-level sanitation potentially offering a broader surveillance snapshot, a closer to real-time monitoring since open drains and rivers may reflect a more recent contamination, and lower infrastructure requirements as it typically requires less investment in specialized infrastructure compared with the collection and analysis of fecal sludge. On the other hand, collecting open drain and river samples can be subjected to dilution for instance from urban runoff with potential subsequent decrease the detection sensitivity. Since it collects water from the surroundings, including agricultural and industrial runoff, it may contain various environmental contaminants that may introduce further inhibitors to the samples that may hinder the molecular detection. This also implies that the water quality in open drains and rivers can be highly variable, depending on factors such as rainfall, seasonal changes, and waste disposal practices, which can affect the consistency and reliability of surveillance data. In LEDCs without proper sewer systems, ES in open drains and rivers can provide broad, low-cost monitoring. However, it requires careful interpretation due to potential dilution and contamination. Despite these challenges, it remains a valuable tool for assessing public health.

Data from the Africa CDC indicated an increase in Dengue and Chikungunya cases in Africa as the year progressed, which aligns with our findings, particularly regarding the co-occurrence of DENV and CHIKV RNA.^27^ Despite no reported cases of DENV, CHIKV, WNV, or USUV in Mozambique in 2023, and the first reported case of CHIKV, in 2023, in Africa occurring only on June 23, our data reveal significant underreporting of these viruses in Mozambique and potentially other African countries. The epidemiology of arboviruses is intricate, encompassing entomological, sociodemographic, and environmental factors like temperature, humidity, precipitation, and hydrology. The diverse climates across the African continent contribute to varied patterns of arboviral infections. In Eastern Africa, arboviral epidemiology is closely linked to rainy seasons, which increase mosquito populations.^28^

Mozambique experiences two primary seasons: the rainy season from October to March, and the dry season from April to September. A recent meta-analysis highlighted that in Eastern Africa, Dengue infections have two distinct peaks: March to May and September to December.^29^ This aligns with our study, where the highest DENV RNA viral load was observed in March 2023. Similarly, CHIKV RNA viral loads peaked in March and April, although its presence was constant throughout the study. A study conducted in Quelimane, Mozambique, indicated endemic transmission of CHIKV rather than outbreaks, which is in agreement with our findings.^30^

While little is known about USUV, a study on WNV infections in horses in South Africa showed most cases occurred from February to April, peaking in March.^31^ Our study found sporadic USUV cases between February and April 2023.^32^ The low prevalence of USUV and absence of WNV detections could be attributed to the low viremia levels caused in humans and non-bird animals as they are considered only dead-end hosts. Our results highlight the value of environmental surveillance in assessing arboviral circulation.

Although no arboviral cases were reported in Mozambique, uncertainties about disease prevalence remain when analyzing raw wastewater and environmental waters. Information on the concentration of viral particles excreted by infected individuals or the levels of these viruses in vectors and sylvatic cycle is sparse and difficult to obtain. Water, including river water, is part of the vectors’ lifecycle. If the larvae degrade and the larvae contain arboviruses, the larvae may release these arboviruses into the river water. Additionally, animals can be reservoirs of arboviruses, many of them being dead-end hosts, producing low levels of viremia and therefore, not participating in the potential infection of mosquitoes, but can still excrete the viruses.^33^ Indeed, our work results are mostly attributed to human activities rather than animal sources, as the study was conducted in an urban area with little presence of wildlife. This reduced the likelihood of signal originated by animals influencing our study. The authors also investigated potential fecal sources, including birds and pigs, in addition to assessing human fecal contamination using PMMoV. They found a significant influence from human fecal contamination, a lesser impact from birds, and an even smaller contribution from pig fecal contamination (data not shown). These fecal sources were selected due to their relevance in the catchment area. A drawback from environmental surveillance is that it cannot intervene at the individual level or distinguish between permanent and transient populations.

Continuous and active surveillance is crucial for timely detection and management of potential arboviral outbreaks. The detection of specific arboviral genomes in environmental waters adds value to traditional syndromic, vector, and sylvatic surveillance methods. Combining environmental water data with clinical samples enhances the ability to predict infection risks and mitigate potential outbreaks. Integrating data on vector occurrence and distribution from environmental waters with coordinated surveillance of *Aedes* and *Culex* mosquitoes is essential for controlling vectorborne diseases.

Further research is needed to understand arboviral excretion levels in symptomatic and asymptomatic individuals, infection patterns, and vector dynamics. This knowledge could enable more accurate modeling of infection levels in wastewater and environmental systems and improve epidemiological predictions.^34,35^ Additionally, tools that differentiate between DENV serotypes and identify CHIKV and USUV clades will provide better insights for health authorities. Despite its limitations, environmental surveillance is poised to become a critical tool in managing arboviral diseases, especially in regions with inadequate surveillance systems. Environmental surveillance provides a near-real-time overview of community-wide virus circulation in the river basin, helping to understand virus dynamics in settings lacking effective clinical information. The promising results from this study also highlight the need to expand ES beyond polio to encompass additional pathogens, such as arboviruses, *Vibrio cholerae*, *Salmonella typhimurium*, and antimicrobial resistance, among others. This is especially important in LEDC where clinical surveillance is often inaccessible.

## Supplementary Material



## Notes

Additional experimental details, materials, and methods, and quality control as well as Tables S1–S6 and Figures S1–S4 (PDF)
