# Supplementary material for: Environmental Surveillance of Vector-Borne Diseases in a Non-Sewered System: A Case Study in Mozambique
Source: Environ Sci Technol. 2025 Feb 14;59(7):3411–21. doi: 10.1021/acs.est.4c09860 (PMC12818743; doi:10.1021/acs.est.4c09860)
Supplement: Supplementary file 1 [file es4c09860_si_001.pdf]

Supporting Information for

Environmental surveillance of vectorborne diseases: a case study in Mozambique

Silvia Monteiro[1,2,3*], Filipa Nunes[1], Nidia Cangi Vaz[4], Clemêncio Nhantumbo[5], Dinis Luiz Juízo[5], and Ricardo Santos[1,2,3*]

[1] Laboratório de Análises, Instituto Superior Técnico, Universidade de Lisboa, 1049-001 Lisboa, Portugal
[2] Civil Engineering Research and Innovation for Sustainability, Instituto Superior Técnico, Universidade de Lisboa, 1049-001 Lisboa, Portugal
[3] Department of Nuclear Sciences and Engineering, Instituto Superior Técnico, Universidade de Lisboa, 1049-001 Lisboa, Portugal
[4] Centro de Biotecnologia, Universidade Eduardo Mondlane, Maputo 1102, Mozambique
[5] Faculdade de Engenharia, Universidade Eduardo Mondlane, Maputo 1102, Mozambique

* Corresponding author. E-mail address: silvia.monteiro@tecnico.ulisboa.pt or ricardosantos@tecnico.ulisboa.pt

Summary: 19 pages, 6 sections, 6 tables, 4 figures

Table of Contents

Supplementary Methods S3

Section S1. Wastewater analysis. S3

Section S2. Porcine epidemic diarrhea virus (PEDV) strain, cell lines and quantification by RT-dPCR. S4

Section S3. Additional details for the Minimum Information guidelines. S4

Section S4. Standard curve performance. S4

Section S5. Additional QA/QC for wastewater samples. S5

Section S6. Calculation of the percentage of positive samples. S5

Table S1. Details of the sampling locations from this study. S6

Table S2. Details on the assays used in this study. S7

Table S3. MIQE checklist. S10

Table S4. List of microorganisms used in this study, and the name of the target gene. S11

Table S5. List and concentrations of the non-target microorganisms S12

Table S6. Load and interquartile range (IQR) of the arboviral targets during the different sampling campaigns S13

Figure S1. Map of Mozambique and the sampling points covered by this study (A). Location of point and non-point sources of contamination (B) S14

Figure S2. Environmental Microbiology Minimum Information (EMMI) guidelines checklist S15

Figure S3. Arboviral RNA concentration in river water samples collected at each sampling location from the Infulene river basin in Maputo, Mozambique. S16

Figure S4. NCBI Multiple Sequence Alignment Viewer for USUV. S18

References S19

Supplementary Methods

Section S1. Wastewater analysis. The moment the samples arrived at the laboratory, they were promptly processed. The river water concentration was carried out following established procedures.[1] A volume of 300 mL of river water from the Infulene river basin, complete with solids, underwent concentration using polyethylene glycol 8000 (PEG 8000). The volume of analysis was chosen due to the likely concentration of these viruses in the chosen river. This decision is supported by Lee et al. (2022), who used mathematical models to evaluate the sensitivity of different volumes for detecting arboviruses in wastewater, showing that larger volumes may be necessary for their detection.[2] The samples were allowed to precipitate with 20% PEG 8000 and followed by overnight incubation at 4 ºC. After precipitation, the samples were subjected to centrifugation at 10,000 xg for 30 minutes. The supernatant was discarded, and the pellet was reconstituted in 5 mL of 1x PBS at a pH of 7.4. These samples were stored at -80 (±10) ºC for subsequent processing. Before freezing the concentrated samples, a 220 µL aliquot was immediately extracted for viral nucleic acids. The extraction of viral nucleic acids was accomplished using the QIAamp FAST DNA Stool Mini kit from QIAGEN in Germany and was then eluted in a final volume of 100 µL. This kit is effective in cleaning up complex matrices, such as stool samples, significantly reducing the interference that can affect q(RT)PCR amplification. Additionally, the kit allows for an efficient extraction of RNA. After extraction, the viral RNA was preserved at -80 ºC for a period between 5 and 14 months.

To specifically identify and quantify viral nucleic acids, four-fold dilutions of each viral extract were concurrently tested alongside crude extracts. This dilution step was implemented to overcome any potential amplification inhibition due to the complexity of the samples. The final volume of the reaction mixture was 15 µL, comprising 800 nM of each primer, 200 nM of probe, and 5 µL of the extracted viral nucleic acid. The primer and probe details can be found in Table S2 and were procured from Eurogentec (Seraing, Belgium).[3-9] The RT-qPCR was carried out using the QuantStudio 5 real-time PCR system (ThermoScientific, USA), with the following cycle conditions: 55 ºC for 10 minutes (for reverse transcription), 95 ºC for 10 minutes, and 45 cycles of amplification at 95 ºC for 15 seconds, and 60 ºC for 1 minute. DENV (FAM), WNV (FAM), and USUV (FAM) were measured separately, while the two target genes for CHIKV were multiplexed (Nsp1 – FAM; E – VIC). Each RT-qPCR run included one positive control for each target (viral RNA/DNA), two negative controls (non-template control (NTC)), and extraction negative controls, which consisted solely of water and lysis buffer. Samples were analyzed in duplicate.

The quantification of viral nucleic acids in the samples was determined using a standard curve with serial dilutions of known concentrations of controls. The quantification of DENV and CHIKV relied on calibration curves using 5- and 10-fold dilutions from the TaqMan Arbovirus Triplex Control Kit (ZIKV/DNV/CHIKV) (ThermoScientific, USA), covering a range from 6.0 to 6.0×10^3 and 1.0 to 1.0×10^4 copies per reaction. Standard curves for WNV and USUV were constructed using gBlocks® Gene Fragments (Integrated DNA Technologies, IA, USA). The gBlocks® Gene Fragments were reconstituted as described by the manufacturer, by adding 1X TE solution, to obtain a final concentration of 10 ng/µL. For precise quantification of the gBlocks® Gene Fragments, digital RT-PCR (RT-dPCR) was employed. The amplification of WNV and USUV was performed using the Luna Universal Probe One-Step RT-qPCR (New England Biolabs, Massachusetts, USA). The 15 µL reaction mixture consisted of 7.5 µL

of 2× RT-PCR buffer, 800 nM of each primer, 200 nM of probe, 0.75 µL of Luna WarmStart® RT Enzyme Mix (20X), 3.48 µL of RNase/DNase-free water, and 3 µL of viral target. The reaction mixture was distributed over the QuantStudio 3D Digital PCR chip (Thermo Fischer Scientific, USA) and the chips were transferred to the QuantStudio 3D Digital PCR thermal cycler (Thermo Fischer Scientific, USA). Amplification was carried out as follows: 55 ºC for 10 minutes, 10 minutes at 96 ºC, 40 cycles of 2 minutes at 60 ºC and 30 seconds at 98 ºC, and a final elongation step for 2 minutes at 60 ºC. The reactions were performed in duplicate.

Section S2. Porcine epidemic diarrhea virus (PEDV) strain, cell lines and quantification by RT-dPCR. The Porcine Epidemic Diarrhea Virus (PEDV) strain CV777, provided by Dr. Gloria Sanchez (IATA-CSIC, Spain), an enveloped virus belongs to the Alphacoronavirus genus and Coronaviridae family, causing porcine epidemic diarrhea. The propagation of PEDV was carried out in the Vero cell line (ATCC CCL-81, LGC Standards, Spain). Vero cells were cultured in Dulbecco's Modified Eagle's Medium (DMEM; Gibco, Waltham, MA USA) supplemented with 100 units/mL penicillin (Lonza, Basel, Switzerland), 100 units/mL streptomycin (Lonza), and 10% heat-inactivated fetal bovine serum (Biological Industries, Kibbutz Beit-Haemek, Israel) in T175 flasks at 37 (± 1) °C under 5% CO_2. For PEDV infection, cells in T25 flasks were inoculated with 100 µL of viral stock. Two hours post-infection, DMEM with 0.3% tryptose phosphate broth, 100 units/mL penicillin (Lonza, Basel, Switzerland), 100 units/mL streptomycin (Lonza, Basel, Switzerland), and 10 µg/µL trypsin were added. Flasks were then incubated at 37 (± 1) °C in 5% CO_2 for 4 days. PEDV was recovered through three freeze/thaw cycles and centrifugation at 1,100 xg for 10 min.

Quantification was done by RT-dPCR using primers and probes from Table S3 (appendix pp 6) after nucleic acid extraction.[9] Amplification of PEDV was carried out using the AgPath-ID One-Step RT-PCR kit (Thermo Fischer Scientific, Waltham, MA USA). The 15 µL reaction mixture included 7.5 µL of 2× RT-PCR buffer, 0.6 µL of 25× RT-PCR enzyme mix, 800 nM of each primer, 200 nM of the probe, 3.63 µL RNase/DNase-free water, and 3 µL of DNA (diluted 4-, 5-, 6- fold). This mixture was evenly distributed over the QuantStudio 3D Digital PCR chip (Thermo Fischer Scientific, Waltham, MA USA), and the chips were then transferred to the QuantStudio 3D Digital PCR thermal cycler. Amplification conditions for PEDV were as follows: 10 min at 45 °C, 10 min at 96 °C, 39 cycles of 2 min at 60 °C and 30 s at 98 °C, and a final elongation step for 2 min at 60 °C. Each reaction was run in duplicate, and a non-template control (NTC) was included in every run. Following absolute quantification by RT-dPCR, a stock solution was prepared in DNase/RNase free water, resulting in a PEDV final concentration of 1.21×10^4 copies/L in wastewater, consistent across all subsequent experiments.

Section S3. Additional details for the Minimum Information guidelines. In addition to the information already presented in the main manuscript and the appendix, there is a need to include supplementary details concerning sampling, sample treatment, and q(RT)PCR. You can find a comprehensive checklist for this in Table S1 and Figure S1.[10,11]

Section S4. Standard curve performance. Amplification efficiencies were 98 ± 1.15%, 102 ± 1.35%, 101 ± 4.41%, 99 ± 2.00%, and 104 ± 2.27%, for DENV, CHIKV Nsp1,

CHIKV E, WNV, and USUV quantification, respectively, with a correlation coefficient of 0.998 ± 0.006, 0.999 ± 0.004, 0.993 ± 4.59 x 10^{-4}, 0.998 ± 0.010, and 0.995 ± 0.007.

S**ection S5. Additional QA/QC for wastewater samples.** For each RT-qPCR assay, we conducted positive, negative (NTC), and both negative and positive extraction controls.

Section S6. Calculation of the percentage of positive samples.

$$\%\ Positive\ samples = \ Total\ \#\ positive\ samples * 100/Total\ \#\ samples$$

Location	GIS	# samples
1	25º55’25”S; 32º32’11”E	7
2	25º55’18”S; 32º32’21”E	7
3	25º55’14”S; 32º32’33”E	6
4	25º55’14”S; 32º32’35”E	7
5	25º55’14”S; 32º32’39”E	8
6	25º54’53”S; 32º32’34”E	7
7	25º49’36”S; 32º33’58”E	8
8	25º49’30”S; 32º34’11”E	8
9	25º46’59”S; 32º34’38”E	8
Total		66

Table S1. Details of the sampling locations from this study. Information on the Geographic Information System (GIS), and the number of samples tested at each sampling point are provided.

Target	Sequence 5' – 3'	Size (bp)	Ref.
DENV Forward	GARAGACCAGAGATCCTGCTGTCT		
Reverse	ACCATTCCATTTTCTGGCGTT	73	3
Probe	AGCATCATTCCAGGCAC		
CHIKV Nsp1 Forward	TGATCCCGACTCAACCATCCT		
Reverse	GGCAAACGCAGTGGTACTTCCT	82	4
Probe	TCCGACATCATCCTCCTTGCTGGC		
CHIKV E1 Forward	TCACTCCCTGTTGGACTTGATAGA		
Reverse	TTGACGAACAGAGTTAGGAACATACC	125	5
Probe	AGGTACGCGCTTCAAGTTCGGCG		
WNV Forward	CAGACCACGCTACGGCG		
Reverse	CTAGGGCCGCGTGGG	103	6
Probe	TCTGCGGAGAGTGCAGTCTGCGAT		
USUV Forward	CAAAGCTGGACAGACATCCCTTAC		
Reverse	CGTAGATGTTTTCAGCCCACGT	103	7
Probe	AAGACATATGGTGTGGAAGCCTGATAGGCA		
MNV Forward	CACGCCACCGATCTGTTCTG		
Reverse	GCGCTGCGCCATCACTC	108	8
Probe	CGCTTTGGAACAATG		
PEDV Forward	CAGGACACATTCTTGGTGGTCTT		
Reverse	CAAGCAATGTACCACTAAGGAGTGTT	140	9
Probe	ACGCGCTTCTCACTAC		

Primers and probes were purchased from Eurogentec (Seraing, Belgium). All probes contained fluorescent molecules and quenchers (5' FAM or HEX/BHQ1 or MGB); DENV, dengue virus; CHIKV, Chikungunya virus; WNV, West Nile virus; USUV, Usutu virus; MNV, murine norovirus; PEDV, porcine epidemic diarrhea virus; HEX, hexachloro-fluorescein; FAM, 6-fluorescein amidite; BHQ1, black hole quencher 1; MGB, minor groove binder.

Table S2. Details on the assays used in this study. Forward and reverse primers and probe sequences used in this study for the detection of viral RNA.

ITEM TO CHECK	IMPORTANCE	CHECKLIST
**EXPERIMENTAL DESIGN**		
Definition of experimental and control groups	**E**	**Y**
Number within each group	E	**Y**
Assay carried out by core lab or investigator's lab?	D	Y
Acknowledgement of authors' contributions	D	Y
**SAMPLE**		
Description	**E**	Y
Volume/mass of sample processed	D	Y
Microdissection or macrodissection	**E**	NA
Processing procedure	**E**	**Y**
If frozen - how and how quickly?	**E**	N
If fixed - with what, how quickly?	**E**	NA
Sample storage conditions and duration (especially for FFPE samples)	**E**	**Y**
**NUCLEIC ACID EXTRACTION**		
Procedure and/or instrumentation	**E**	Y
Name of kit and details of any modifications	**E**	Y
Source of additional reagents used	D	Y
Details of DNase or RNAse treatment	**E**	NOT PERFORMED
Contamination assessment (DNA or RNA)	**E**	NOT PERFORMED
Nucleic acid quantification	**E**	NOT PERFORMED
Instrument and method	**E**	NOT PERFORMED
Purity (A260/A280)	D	NA
Yield	D	NA
RNA integrity method/instrument	**E**	NA
RIN/RQI or Cq of 3' and 5' transcripts	**E**	NA
Electrophoresis traces	D	NA
Inhibition testing (Cq dilutions, spike or other)	**E**	Y
**REVERSE TRANSCRIPTION**		
Complete reaction conditions	**E**	Y
Amount of RNA and reaction volume	**E**	Amount of RNA not measured but reaction volume provided
Priming oligonucleotide (if using GSP) and concentration	**E**	Y
Reverse transcriptase and concentration	**E**	Y
Temperature and time	**E**	Y
Manufacturer of reagents and catalogue numbers	D	NA
Cqs with and without RT	D	NA
Storage conditions of cDNA	D	NA
**qPCR TARGET INFORMATION**		
If multiplex, efficiency and LOD of each assay.	**E**	Y
Sequence accession number	**E**	Y
Location of amplicon	D	N
Amplicon length	**E**	Y
In silico specificity screen (BLAST, etc.) Pseudogenes, retropseudogenes or otherhomologs?	**E**	N

Sequence alignment	D	N
	D	N
Secondary structure analysis of amplicon	D	N
Location of each primer by exon or intron (if applicable)	**E**	NA
What splice variants are targeted?	**E**	NA
**qPCR OLIGONUCLEOTIDES**		
Primer sequences	**E**	Y
RTPrimerDB Identification Number	D	N
Probe sequences	D	Y
Location and identity of any modifications	**E**	NA
Manufacturer of oligonucleotides	D	Y
Purification method	D	N
**qPCR PROTOCOL**		
Complete reaction conditions	**E**	Y
Reaction volume and amount of cDNA/DNA	**E**	Y
Primer, (probe), Mg++ and dNTP concentrations	**E**	Y
Polymerase identity and concentration	**E**	PROPRIETARY
Buffer/kit identity and manufacturer	**E**	Y
Exact chemical constitution of the buffer	D	PROPRIETARY
Additives (SYBR Green I, DMSO, etc.)	**E**	N
Manufacturer of plates/tubes and catalog number	D	N
Complete thermocycling parameters	**E**	Y
Reaction setup (manual/robotic)	D	N
Manufacturer of qPCR instrument	**E**	Y
**qPCR VALIDATION**		
Evidence of optimization (from gradients)	D	N
Specificity (gel, sequence, melt, or digest)	**E**	N
For SYBR Green I, Cq of the NTC	**E**	NA
Standard curves with slope and y-intercept	**E**	
PCR efficiency calculated from slope	**E**	Y
Confidence interval for PCR efficiency or standard error	D	N
r2 of standard curve	**E**	Y
Linear dynamic range	**E**	Y
Cq variation at lower limit	**E**	N
Confidence intervals throughout range	D	N
Evidence for limit of detection	**E**	Y
If multiplex, efficiency and LOD of each assay.	**E**	Y
**DATA ANALYSIS**		
qPCR analysis program (source, version)	**E**	N
Cq method determination	**E**	Y
Outlier identification and disposition	**E**	Y
Results of NTCs	**E**	Y
Justification of number and choice of referencegenes	**E**	N
Description of normalization method	**E**	Y
Number and concordance of biological replicates	D	Y

Number and stage (RT or qPCR) of technical replicates	**E**	Y
Repeatability (intra-assay variation)	E	N
Reproducibility (inter-assay variation, %CV)	D	N
Power analysis	D	N
Statistical methods for result significance	**E**	Y
Software (source, version)	E	Y
Cq or raw data submission using RDML	**D**	N

Table S3. MIQE checklist. All essential information (E) must be submitted with the manuscript if/when performed. Desirable information (D) should be submitted if available. If using primers obtained from RTPrimerDB, information on qPCR target, oligonucleotides, protocols and validation are available from that source.[10]

Virus	Region target	Non-target testing (negatives)
Dengue virus (DENV)	3' untranslated region	Intact *Escherichia coli* (ATCC 25922); intact *Enterococcus faecalis* (Enterococcus faecalis (ATCC 19433); intact adenovirus 3 (ATCC VR-3) and 41 (ATCC VR-930); intact Aichi virus (kindly provided by Dr. Hata from the University of Toyama); intact enterovirus (coxsackievirus B3 kindly provided by Dr. Francisco Lucena from the University of Barcelona); gRNA Hepatitis A virus (Hepatitis A virus – Q standard, Ceeram); g RNA Hepatitis E virus (Hepatitis E virus – Q standard, Ceeram); gRNA norovirus GI (norovirus GI – Q standard, Ceeram) and GII (norovirus GII – Q standard, Ceeram); gRNA rotavirus (rotavirus – Q standard, Ceeram); oocysts *Cryptosporidium* spp. (kindly provided by Moredun Scientific)
Chikungunya virus (CHIKV)	Non-structural protein 1	
	Envelope	
West Nile virus (WNV)	Non-structural protein 1	
Usutu virus	Non-structural protein 5	

Table S4. List of microorganisms used in this study, and the name of the target gene. The list of non-target controls tested to determine the specificity are provided. ATCC – American Type Culture Collection.

Viral target	Non-target microorganisms – concentration (copies/reaction)
DENV<br>CHIKV Nsp1<br>CHIKV E<br>WNV<br>USUV	*Escherichia coli* – 3.3×10^1 and 3.3×10^7
	Enterococcus faecalis – 5.5×10^1 and 5.5×10^7
	Aichi virus – 1.5×10^1 and 1.5×10^6
	Adenovirus 3 – 2.2×10^1 and 2.2×10^6
	Adenovirus 41 – 3.4×10^1 and 3.4×10^6
	Enterovirus – 3.9×10^1 and 3.9×10^6
	Hepatitis A virus – 1.0×10^1 and 1.0×10^6
	Hepatitis E virus – 2.2×10^1 and 2.2×10^6
	Norovirus GI - 1.0×10^1 and 1.0×10^6
	Norovirus GII - 1.0×10^1 and 1.0×10^6
	Rotavirus – 7.0×10^1 and 7.0×10^5
	Cryptosporidium parvum – 1.0×10^1 and 1.0×10^6

Table S5. List and concentrations of the non-target microorganisms, commonly found in raw wastewater, used to test the specificity of the arboviral assays used in this study.

Sampling campaign	DENV	CHIKV Nsp1	CHIKV E	USUV
02.17.2023	0 (0-4.5 x 10^5)	2.4 x 10^5 (1.4 x 10^5-7.1 x 10^5)	1.2 x 10^6 (8.2 x 10^5-3.1 x 10^6)	0 (0-3.5 x 10^2)
03.17.2023	3.8 x 10^5 (2.7 x 10^{-5}-4.6 x 10^5)	5.2 x 10^4 (4.3 x 10^4-1.0 x 10^5)	4.9 x 10^5 (3.3 x 10^5-1.0 x 10^6)	0 (0-0)
04.25.2023	0 (0-0)	3.5 x 10^5 (0-9.8 x 10^5)	1.3 x 10^6 (3.3 x 10^5-2.3 x 10^6)	0 (0-0)
05.11.2023	1.0 x 10^5 (7.5 x 10^4-5.5 x 10^5)	5.8 x 10^5 (1.9 x 10^5-7.3 x 10^5)	3.2 x 10^5 (1.5 x 10^5-4.9 x 10^5)	0 (0-0)
06.15.2023	6.5 x 10^5 (3.3 x 10^5-8.8 x 10^5)	9.8 x 10^4 (4.9 x 10^4-2.6 x 10^5)	4.4 x 10^4 (0-5.6 x 10^5)	0 (0-0)
07.13.2023	2.7 x 10^5 (2.0 x 10^5-9.9 x 10^5)	1.6 x 10^5 (1.4 x 10^5-2.5 x 10^5)	1.4 x 10^6 (3.5 x 10^5-2.1 x 10^6)	0 (0-0)
08.10.2023	2.5 x 10^5 (1.1 x 10^5-7.2 x 10^5)	1.4 x 10^5 (6.4 x 10^4-1.8 x 10^5)	6.2 x 10^5 (2.0 x 10^5-1.1 x 10^6)	0 (0-0)
09.06.2023	1.2 x 10^5 (6.1 x 10^4-1.6 x 10^5)	7.5 x 10^5(5.9 x 10^5-8.1 x 10^5)	3.6 x 10^5 (1.6 x 10^5-7.7 x 10^5)	0 (0-0)0

Table S6. Load and interquartile range (IQR) of the arboviral targets during the different sampling campaigns.

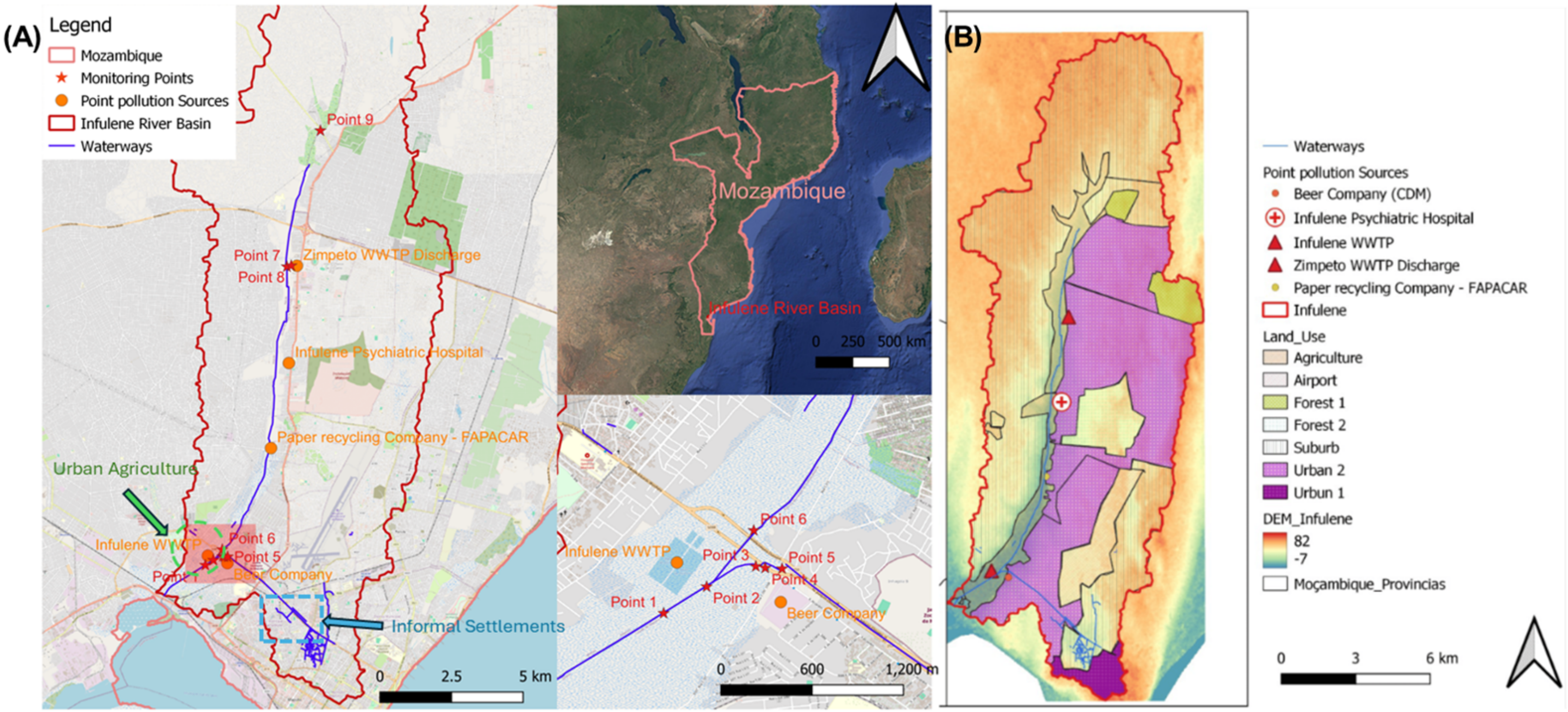

Figure S1. Map of Mozambique and the sampling points covered by this study (A). Location of point and non-point sources of contamination (B)[11]

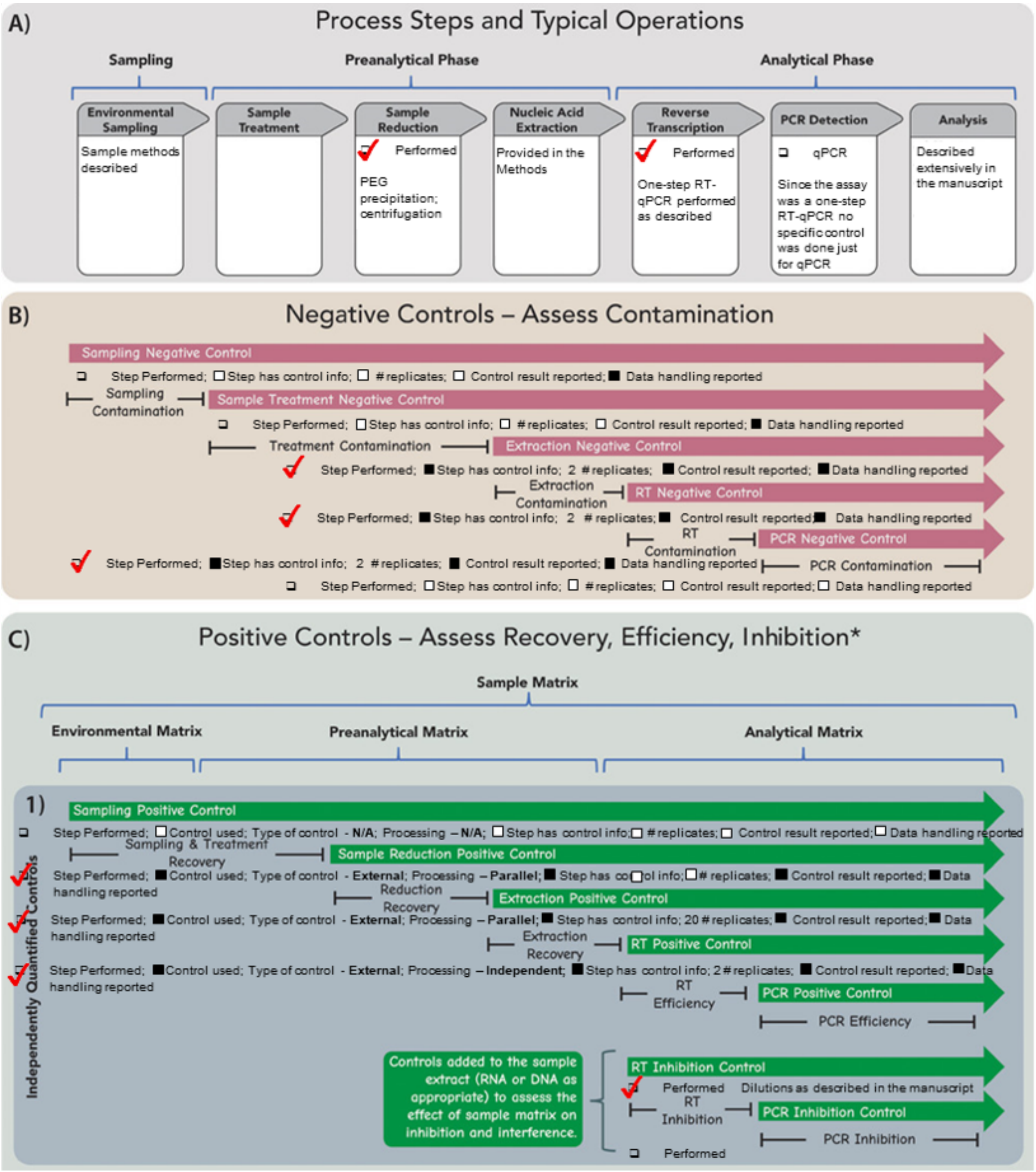

Figure S2. Environmental Microbiology Minimum Information (EMMI) guidelines checklist [12]

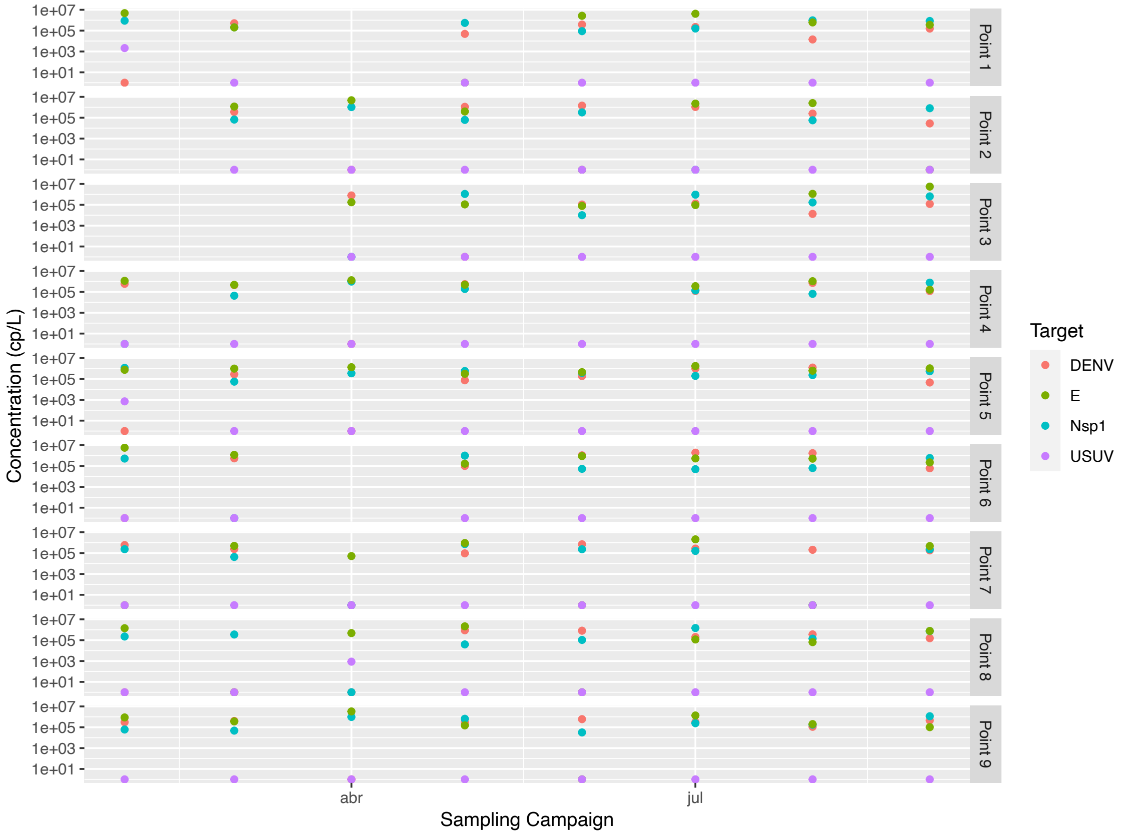

Figure S3. Arboviral RNA concentration in river water samples collected at each sampling location from the Infulene river basin in Maputo, Mozambique.

Report of monthly measurements. Each grid represents one sampling point collected throughout the sampling campaign. Measurements were conducted between February and September 2023. DENV = Dengue virus. E = Chikungunya virus *E1* gene. Nsp1 = chikungunya virus *Nsp1* gene. USUV = Usutu virus.

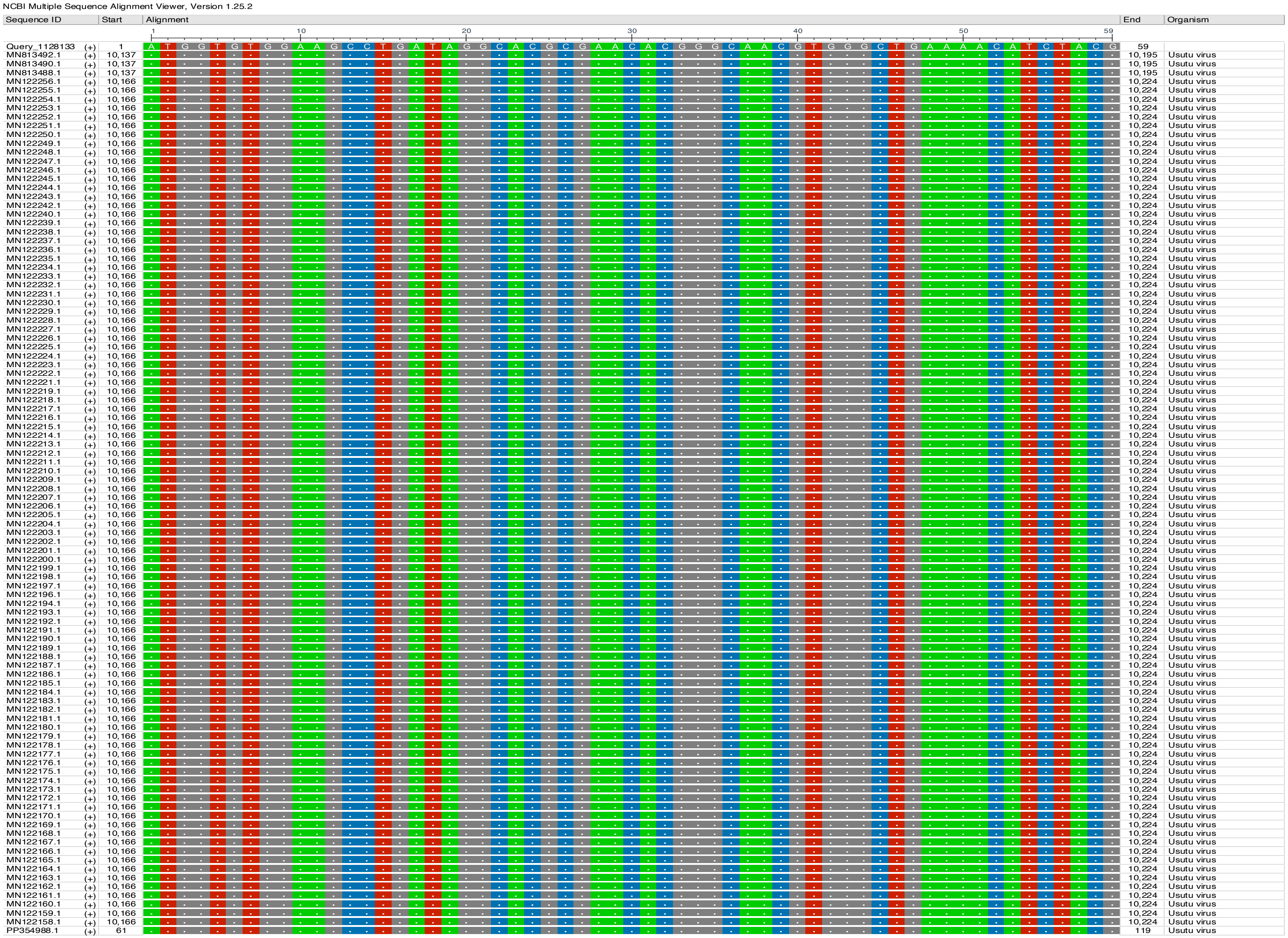
NCBI Multiple Sequence Alignment Viewer, Version 1.25.2
Sequence ID
Start
Alignment
End
Organism
1 10 20 30 40 50 59
Query_1128133 (+) 1 ATGGTGTGGAAGCCTGATAGGCACGCGAACACGGGCAACGTGGGCTGAAAACATCTACG 59
MN813492.1 (+) 10,137 10,195 Usutu virus
MN813490.1 (+) 10,137 10,195 Usutu virus
MN813488.1 (+) 10,137 10,195 Usutu virus
MN122256.1 (+) 10,166 10,224 Usutu virus
MN122255.1 (+) 10,166 10,224 Usutu virus
MN122254.1 (+) 10,166 10,224 Usutu virus
MN122253.1 (+) 10,166 10,224 Usutu virus
MN122252.1 (+) 10,166 10,224 Usutu virus
MN122251.1 (+) 10,166 10,224 Usutu virus
MN122250.1 (+) 10,166 10,224 Usutu virus
MN122249.1 (+) 10,166 10,224 Usutu virus
MN122248.1 (+) 10,166 10,224 Usutu virus
MN122247.1 (+) 10,166 10,224 Usutu virus
MN122246.1 (+) 10,166 10,224 Usutu virus
MN122245.1 (+) 10,166 10,224 Usutu virus
MN122244.1 (+) 10,166 10,224 Usutu virus
MN122243.1 (+) 10,166 10,224 Usutu virus
MN122242.1 (+) 10,166 10,224 Usutu virus
MN122240.1 (+) 10,166 10,224 Usutu virus
MN122239.1 (+) 10,166 10,224 Usutu virus
MN122238.1 (+) 10,166 10,224 Usutu virus
MN122237.1 (+) 10,166 10,224 Usutu virus
MN122236.1 (+) 10,166 10,224 Usutu virus
MN122235.1 (+) 10,166 10,224 Usutu virus
MN122234.1 (+) 10,166 10,224 Usutu virus
MN122233.1 (+) 10,166 10,224 Usutu virus
MN122232.1 (+) 10,166 10,224 Usutu virus
MN122231.1 (+) 10,166 10,224 Usutu virus
MN122230.1 (+) 10,166 10,224 Usutu virus
MN122229.1 (+) 10,166 10,224 Usutu virus
MN122228.1 (+) 10,166 10,224 Usutu virus
MN122227.1 (+) 10,166 10,224 Usutu virus
MN122226.1 (+) 10,166 10,224 Usutu virus
MN122225.1 (+) 10,166 10,224 Usutu virus
MN122224.1 (+) 10,166 10,224 Usutu virus
MN122223.1 (+) 10,166 10,224 Usutu virus
MN122222.1 (+) 10,166 10,224 Usutu virus
MN122221.1 (+) 10,166 10,224 Usutu virus
MN122219.1 (+) 10,166 10,224 Usutu virus
MN122218.1 (+) 10,166 10,224 Usutu virus
MN122217.1 (+) 10,166 10,224 Usutu virus
MN122216.1 (+) 10,166 10,224 Usutu virus
MN122215.1 (+) 10,166 10,224 Usutu virus
MN122214.1 (+) 10,166 10,224 Usutu virus
MN122213.1 (+) 10,166 10,224 Usutu virus
MN122212.1 (+) 10,166 10,224 Usutu virus
MN122211.1 (+) 10,166 10,224 Usutu virus
MN122210.1 (+) 10,166 10,224 Usutu virus
MN122209.1 (+) 10,166 10,224 Usutu virus
MN122208.1 (+) 10,166 10,224 Usutu virus
MN122207.1 (+) 10,166 10,224 Usutu virus
MN122206.1 (+) 10,166 10,224 Usutu virus
MN122205.1 (+) 10,166 10,224 Usutu virus
MN122204.1 (+) 10,166 10,224 Usutu virus
MN122203.1 (+) 10,166 10,224 Usutu virus
MN122202.1 (+) 10,166 10,224 Usutu virus
MN122201.1 (+) 10,166 10,224 Usutu virus
MN122200.1 (+) 10,166 10,224 Usutu virus
MN122199.1 (+) 10,166 10,224 Usutu virus
MN122198.1 (+) 10,166 10,224 Usutu virus
MN122197.1 (+) 10,166 10,224 Usutu virus
MN122196.1 (+) 10,166 10,224 Usutu virus
MN122194.1 (+) 10,166 10,224 Usutu virus
MN122193.1 (+) 10,166 10,224 Usutu virus
MN122192.1 (+) 10,166 10,224 Usutu virus
MN122191.1 (+) 10,166 10,224 Usutu virus
MN122190.1 (+) 10,166 10,224 Usutu virus
MN122189.1 (+) 10,166 10,224 Usutu virus
MN122188.1 (+) 10,166 10,224 Usutu virus
MN122187.1 (+) 10,166 10,224 Usutu virus
MN122186.1 (+) 10,166 10,224 Usutu virus
MN122185.1 (+) 10,166 10,224 Usutu virus
MN122184.1 (+) 10,166 10,224 Usutu virus
MN122183.1 (+) 10,166 10,224 Usutu virus
MN122182.1 (+) 10,166 10,224 Usutu virus
MN122181.1 (+) 10,166 10,224 Usutu virus
MN122180.1 (+) 10,166 10,224 Usutu virus
MN122179.1 (+) 10,166 10,224 Usutu virus
MN122178.1 (+) 10,166 10,224 Usutu virus
MN122177.1 (+) 10,166 10,224 Usutu virus
MN122176.1 (+) 10,166 10,224 Usutu virus
MN122175.1 (+) 10,166 10,224 Usutu virus
MN122174.1 (+) 10,166 10,224 Usutu virus
MN122173.1 (+) 10,166 10,224 Usutu virus
MN122172.1 (+) 10,166 10,224 Usutu virus
MN122171.1 (+) 10,166 10,224 Usutu virus
MN122170.1 (+) 10,166 10,224 Usutu virus
MN122169.1 (+) 10,166 10,224 Usutu virus
MN122168.1 (+) 10,166 10,224 Usutu virus
MN122167.1 (+) 10,166 10,224 Usutu virus
MN122166.1 (+) 10,166 10,224 Usutu virus
MN122165.1 (+) 10,166 10,224 Usutu virus
MN122164.1 (+) 10,166 10,224 Usutu virus
MN122163.1 (+) 10,166 10,224 Usutu virus
MN122162.1 (+) 10,166 10,224 Usutu virus
MN122161.1 (+) 10,166 10,224 Usutu virus
MN122160.1 (+) 10,166 10,224 Usutu virus
MN122159.1 (+) 10,166 10,224 Usutu virus
MN122158.1 (+) 10,166 10,224 Usutu virus
PP354988.1 (+) 61 119 Usutu virus

Figure S4. NCBI Multiple Sequence Alignment Viewer for USUV. The alignment contains only a part of the fragment sequenced for better visualization of the alignment. Each color represents a different nucleotide

References

1 Monteiro, S. et al. A wastewater-based epidemiology tool for COVID-19 surveillance in Portugal. *Sci Total Environ* **804**, 150264 (2022)

2 Lee, W. L. et al. Monitoring human arboviral diseases through wastewater surveillance: Challenges, progress and future opportunities. *Water Res* **223**, 118904 (2022)

3 Gurukumar, K. R. et al. Development of a real time PCR for detection and quantification of Dengue viruses. *Virol J* **6**, 10 (2009)

4 Panning, M., Grywna, K., van Esbroeck, M., Emmerich, P. & Drosten, C. Chikungunya fever in travelers returning to Europe from the Indian Ocean region, 2006. *Emerg Infect Dis* **14(3)**, 416-422 (2008)

5 Lanciotti, R. S. et al. Chikungunya virus in US travelers returning from India, 2006. *Emerg Infect Dis* **13(5)**, 764-767 (2007)

6 Lanciotti, R. D. et al. Rapid detection of west nile virus from human clinical specimens, field-collected mosquitos, and avian samples by a TaqMan reverse transcriptase-PCR assay. *J Clin Microbiol* **38(11)**, 4066-4071 (2000)

7 Nikolay, B., Weidmann, M., Dupressoir, A., Faye, O., Boye, C. S., Diallo, M. & Sall, A. A. Development of a Usutu virus specific real-time reverse transcription PCR assay based on sequenced strains from Africa and Europe. *J Virol Methods* **197**, 51-54 (2014)

8 Baert, L., Wobus, C. E., Van Coillie, E., Thackray, L. B., Debevere, J. & Uyttendaele, M. Detection of murine norovirus 1 using plaque assay, transfection assay, and real-time reverse-transcription-PCR before and after heat exposure. *Appl Environ Microbiol* **74(2)**, 543-546 (2008)

9 Zhou, X. et al. Comparison and evaluation of conventional RT-PCR SYBR green I and TaqMan real-time RT-PCR assays for the detection of porcine epidemic diarrhea virus. *Mol. Cell. Probes* **33**, 36-41 (2017)

10 Austin, S. A. et al. The MIQE guidelines: minimum information for publication of quantitative real-time PCR experiments. *Clin Chem* **55**, 611-622 (2009)

11 Google Earth. earth.google.com/web (last accessed: 26 July 2024)

12 Borchardt, M. A., Boehm, A. B., Salit, M., Spence, S. K., Wigginton, K. R. & Noble R. T. The Environmental Microbiology Minimum Information (EMMI) guidelines: qPCR and dPCR quality and reporting for environmental microbiology. *Environ. Sci. Technol.* **55(15)**,10210-10223 (2021)
